# Short-form development of the specific module of the QLICD-CRF(V2.0) for assessing the quality of life of patients with chronic renal failure

**DOI:** 10.1186/s12874-022-01766-8

**Published:** 2022-11-08

**Authors:** Zhengqin Xiao, Yuxi Liu, Daniel Yee-Tak Fong, Xinping Huang, Min Weng, Chonghua Wan

**Affiliations:** 1 School of Humanities and Management, Research Center for Quality of Life and Applied Psychology, Key Laboratory for Quality of Life and Psychological Assessment and Intervention, Guangdong Medical University, Dongguan, 523808 China; 2 School of Nursing, The University of Hong Kong, Hong Kong, China; 3 Dongguan Enghth’ Hospital, Dongguan, 523325 China; 4 The First Affiliated Hospital of Kunming Medical University, Kunming, 650100 China

**Keywords:** Scale reduction, Generalizability theory, Classical test theory, Item response theory, Optimal test assembly

## Abstract

**Background:**

A short instrument would enhance the viability of a study. Therefore, we aimed to shorten the specific module (SPD-10) of the Quality of Life Instrument for Chronic Diseases - Chronic Renal Failure (QLICD-CRF) for assessing the quality of life of patients with chronic renal failure.

**Methods:**

The 10-item SPD-10 was self-administered to 164 patients with chronic renal failure. A shortened form was first obtained by a tandem use of the classical test theory (CTT), the generalizability theory (GT), and the item response theory (IRT). In addition, we also shortened the SPD-10 by the Optimal Test Assembly (OTA).

**Results:**

Both the tandem use of GT, CTT and IRT, and the OTA derived the same 7-item shortened version (SPD-7). It included items CRF1, CRF2, CRF3, CRF4, CRF6, CRF8, and CRF9 of the SPD-10. The SPD-7 had a Cronbach alpha of 0.78. The correlation coefficients of its total and factor scores with those of the SPD-10 were 0.96 and 0.98, respectively. Confirmatory factor analysis confirmed the unidimensional structure of the SPD-7, with the comparative fit index=0.96, the Tucker-Lewis index=0.94, and the root mean square error of approximation=0.09.

**Conclusion:**

The short-form SPD-7 is reliable and valid for assessing the impact of clinical symptoms and side effects on the quality of life of patients with chronic renal failure. It is an efficient option without compromising the measurement performance of the SPD-10.

## Background

Chronic renal failure (CRF) is an irreversible deterioration of renal function, which has become a major public health problem worldwide [1]. Its clinical manifestations, such as anemia, uremia, hypertension, anorexia, and pruritus, can cause severe physical discomfort to the patients [2]. Moreover, the long-term treatment of CRF renders patients prone to the development of depression, anxiety, and other psychiatric problems [2, 3]. Consequently, CRF patients may have deteriorated quality of life (QOL).

To meet the urgent need for proper evaluation of the QOL of CRF patients in China, the Quality of Life Instrument for Chronic Diseases-Chronic Renal Failure (QLICD-CRF) was developed [4, 5]. The latest version, the 38-item QLICD-CRF V2.0, included a 28-item general module (QLICD-GM V2.0) that applies to patients with any chronic diseases, and a 10-item module specific for CRF patients (SPD-10). The instrument has demonstrated good reliability and validity for measuring the QOL of Chinese CRF patients [4, 5]. However, the instrument is lengthy, which adds a substantial administrative burden and reduces data quality, especially when it is applied in outpatient or community settings. Therefore, it is important to obtain a short-form without compromising its reliability and validity.

There have been several common instrument shortening approaches, including the Generalizability Theory (GT), the Classical Test Theory (CTT) and the Item Response Theory (IRT). The GT determines an appropriate number of items of an instrument by estimating the dependability of scores with the number of items in different forms [6–8]. However, GT does not select items in a shortened form. Thus, the GT method is often used with an item selection strategy. The CTT and IRT methods have been the most popular item selection methods. A CTT model assumes a subject’s observed score is a composite of a true score and measurement error [9–11]. In contrast, IRT directly models an item’s response on its corresponding latent trait and derives measurement properties that are sample independent [12, 13]. To take advantage of the strengths of the three methods, a tandem approach can be adopted [14, 15].

More recently, an alternative scale reduction approach, called the Optimal Test Assembly (OTA), was proposed [16, 17]. The OTA is a mixed-integer programming procedure that uses an estimated IRT model to select a subset of items that best satisfies a pre-specified set of constraints. It can be described as a two-stage process. Stage one searches for the set of items that maximizes the test information for each fixed number of items. In stage two, the minimal set of items that meets the pre-specified criteria would be taken as the optimal short-form [18]. Examples of the criteria are having a Cronbach alpha of at least 95% of that of the full-form and a correlation of at least 0.95 with the full-form. Thus, given the same set of criteria, the short-form obtained by OTA is highly reproducible [16, 17, 19].

Consequently, we attempted to develop a short-form of the SPD-10 domain in the QLICD-CRF to facilitate effective assessment of the QOL in CRF patients, by a tandem use of the three test theories, and the OTA.

## Methods

### Sample

We recruited subjects aged 14 years or older who were (1) diagnosed with CRF; and (2) able to read Simplified Chinese. Subjects who had disturbance of consciousness, cognitive impairment, or other mental illness were excluded. Patients were recruited at the Department of Nephrology of the First Affiliated Hospital of the Kunming Medical University in China.

Eligible patients were explained the study procedures before they were invited to sign a consent form. A total of 164 patients with CRF consented to their study participation. The study protocol and consent form were approved by the Institutional Review Board of the investigators’ affiliated institution.

### Measures

The SPD-10 included 10 items on the frequency of symptoms or severity of side effects in the past week, with each item responding from 1 ("extraordinary") to 5 ("none at all"). Table 1 shows the 10 items of the SPD-10. The total score was standardized in the range from 0 to 100. A higher score indicated milder clinical symptoms and better quality of life [4, 5, 20].

**Table 1:** Item information of the SPD-10

Item	Content description
CRF1	Have you had chest tightness, shortness of breath, or cough with pink foam sputum
CRF2	Have you had feel dizzy or pale
CRF3	Have you had edema of eyelids or face or lower limbs
CRF4	Have you had cramps in your limbs
CRF5	Have you had skin itchiness
CRF6	Have you had sleep wrong (do not sleep at night, want to sleep during the day)
CRF7	Have you urinated a lot at night
CRF8	Have you had muscle or joint pain
CRF9	Have you had decreased urine output
CRF10	Have you had constipation

### Statistical analysis

#### Unidimensionality of the SPD-10

The unidimensionality of the SPD-10 was assessed by confirmatory factor analysis (CFA).

Specifically, we fitted a one-factor model and obtained the comparative fit index (CFI), the Tucker-Lewis index (TLI), and the root mean square error of approximation (RMSEA). If the CFI and TLI ≥ 0.90 and the RMSEA ≤ 0.01, we concluded the one-factor model of the SPD-10 was acceptably and thus unidimensional [21].

### Scale reduction by tandem use of GT, CTT and IRT

The GT encompassed the Generalizability Study (G-study) and the Decision Study (D-study). The G-study estimated the variance components of the measurement error of the test score due to various sources by using the analysis of variance (ANOVA) [11, 22]. The D-study accounted all sources of error when assessing the generalizability of the test score for a fixed number of items [10, 23].

Specifically, in the G-study, we considered the SPD-10 test score as a fixed facet and the 10 items as random by assuming they were randomly selected indicators from an underlying pool. Using the ANOVA, the variance components due to persons, items, and person by item interaction were estimated. In the D-study, we obtained the generalizability coefficient (*G*), the reliability index (*φ*), the relative error variance (${\upsigma}_{\updelta}^2$) and the absolute error variance (${\upsigma}_{\Delta }^2$) for a fixed number of items from 3 to 10. The smallest number of items that had *G* and *φ* > 0.70, and ${\upsigma}_{\updelta}^2$ and ${\upsigma}_{\Delta }^2$ < 0.20 was considered the required number of items in the short-form [10].

Then, we selected the items that satisfied the highest number of criteria based on estimates obtained from the CTT and IRT. The criteria were (1) an item-total correlation coefficient ≥ 0.4; (2) a reduced Cronbach alpha coefficient after deleting the item; (3) a corrected item-total correlation (CITC) ≥ 0.4 [24, 25]; (4) a factor loading ≥ 0.4 in the one-factor CFA [22, 26]; (5) an item discrimination parameter (*α*) estimate > 0.5; (6) estimates of all the threshold parameters (*β*) fell between -4 and 4 [27]; and (7) an average item information ≥ 0.5, which was roughly equivalent to the classical reliability estimate of 0.8.

When multiple sets of items satisfied the aforementioned criteria, the one with the highest average test information was selected [16].

### Scale reduction by OTA

We have also used OTA to search for an optimal reduced set of items without compromising the precision, reliability, and concurrent validity. First, for a fixed number of items from 3 to 10, we used a branch and bound algorithm to search for the set of items that maximized the test information function (TIF) anchored at five points (-3, -1, 0, 1, 3) [18, 19]. The TIF was obtained based on a GPCM. We considered a minimum of three items to ensure a single factor model was identifiable [28]. Thus, eight candidate short-forms were obtained. Second, the final short-form was identified based on the following criteria: (1) a Cronbach alpha at least 95% of that of the SPD-10; and (2) a correlation with both the total and factor scores of the SPD-10 of at least 0.95. These criteria were set to ensure that the short-form would closely resemble the test precision, reliability, and validity of the full-form. The shortest form that fulfilled the criteria was chosen as the final shortened version.

### Statistical software

The CFA was conducted in Mplus7 [29]. The GT analysis was conducted using mGENOVA [30, 31]. The CTT analysis was conducted in SPSS 24.0. The GPCM was fitted using the ltm package in RStudio4.0.5 [32]. The OTA analysis was conducted using the lpSolveAPI package in RStudio4.0.5 [33]. All other data analyses were analyzed using RStudio4.0.5 [34].

## Results

### Characteristics of the sample and the SPD-10

A total of 164 hospitalized patients with CRF consented to participate in our study. Their mean age was 45.6 (standard deviation [SD] = 14.9, range = 14 to 81 years). Of which, 97 (59.1%) were male, 128 (78.0%) were ethnic Han, and 138 (84.1%) were married. Eighty-four (51.2%) patients had poor economic income, 62 (37.8%) were farmers, and 48 (29.3%) attended primary school only.

### Unidimensionality of the SPD-10

The one-factor model of the SPD-10 was adequate in the CFA, with the CFI = 0.95, TLI = 0.94, and RMSEA = 0.07. This indicated the SPD-10 was unidimensional.

### Scale reduction by a tandem use of GT, CTT and IRT

**Fig. 1:**
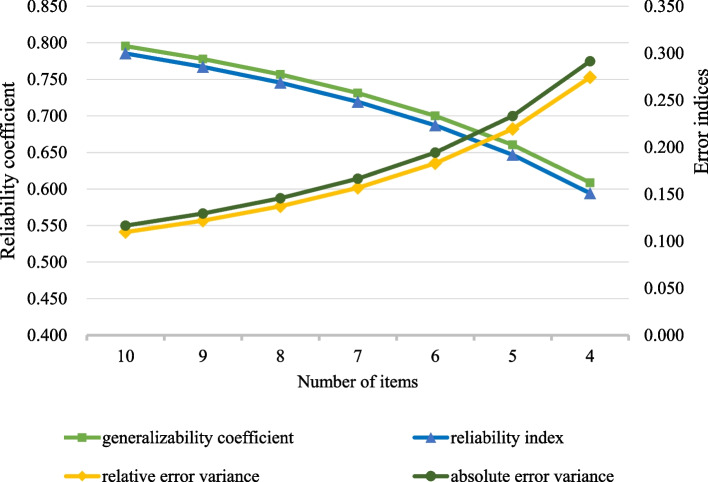
The D-study indices by the number of items of the SPD-10

Figure 1 depicts the indices of the D-study in the GT analysis by each number of items of the SPD-10. A minimum of seven items were required to have the *G* and *ɸ* above 0.70, and the ${\sigma}_{\delta}^2$ and ${\sigma}_{\Delta }^2$ lower than 0.20. Table 2 shows the scaling properties of the 10 items of the SPD-10 by the CTT and IRT analyses. Except for item CRF7, all items had the corrected item-total correlation, Cronbach alpha, item-total correlation, and factor loadings met the predetermined standards. Most items had an average item information lower than 0.50. Overall, the seven items that satisfied the highest number of criteria were CRF1, CRF2, CRF3, CRF4, CRF6, CRF8, and CRF9.

**Table 2:** Scaling information of the SPD-10 items based on the classical test theory (CTT) and item response theory analyses

Item	CTT				Generalized Partial Credit Model						Number of criteria satisfied
	①	②	③	④	①	②	③	④	⑤	⑥	
CRF1	0.563**	0.781	0.435	0.566	0.704	-2.061	-0.731	-2.360	-0.049	0.425	6
CRF2	0.639**	0.773	0.511	0.639	0.789	-1.743	-0.280	-1.199	1.858	0.489	6
CRF3	0.674**	0.770	0.531	0.644	0.707	-0.921	-0.177	-0.862	0.615	0.480	6
CRF4	0.571**	0.777	0.476	0.583	0.661	-1.055	-1.790	-1.351	0.850	0.399	6
CRF5	0.582**	0.777	0.477	0.569	0.572	-0.488	-1.669	-1.461	0.168	0.353	6
CRF6	0.620**	0.768	0.558	0.645	0.987	-1.681	-0.915	-1.594	0.190	0.682	7
CRF7	0.338**	0.808	0.192	0.238	0.194	-2.969	-2.959	-0.955	3.232	0.050	1
CRF8	0.609**	0.774	0.500	0.579	0.686	-1.755	-1.207	-0.831	1.453	0.402	6
CRF9	0.675**	0.767	0.549	0.661	0.849	-0.831	-0.921	-0.414	1.227	0.581	7
CRF10	0.585**	0.779	0.456	0.523	0.522	-1.094	-1.381	-0.912	0.268	0.304	6

CTT: ① Item-total correlation, ② Cronbach alpha, ③ Corrected item-total correlation, ④ Factor loading; GPCM: ① *α,* ②~⑤ *β*_1_~*β*_4_, ⑥ Average information. **P<0.01

### Scale reduction by OTA

**Fig. 2:**
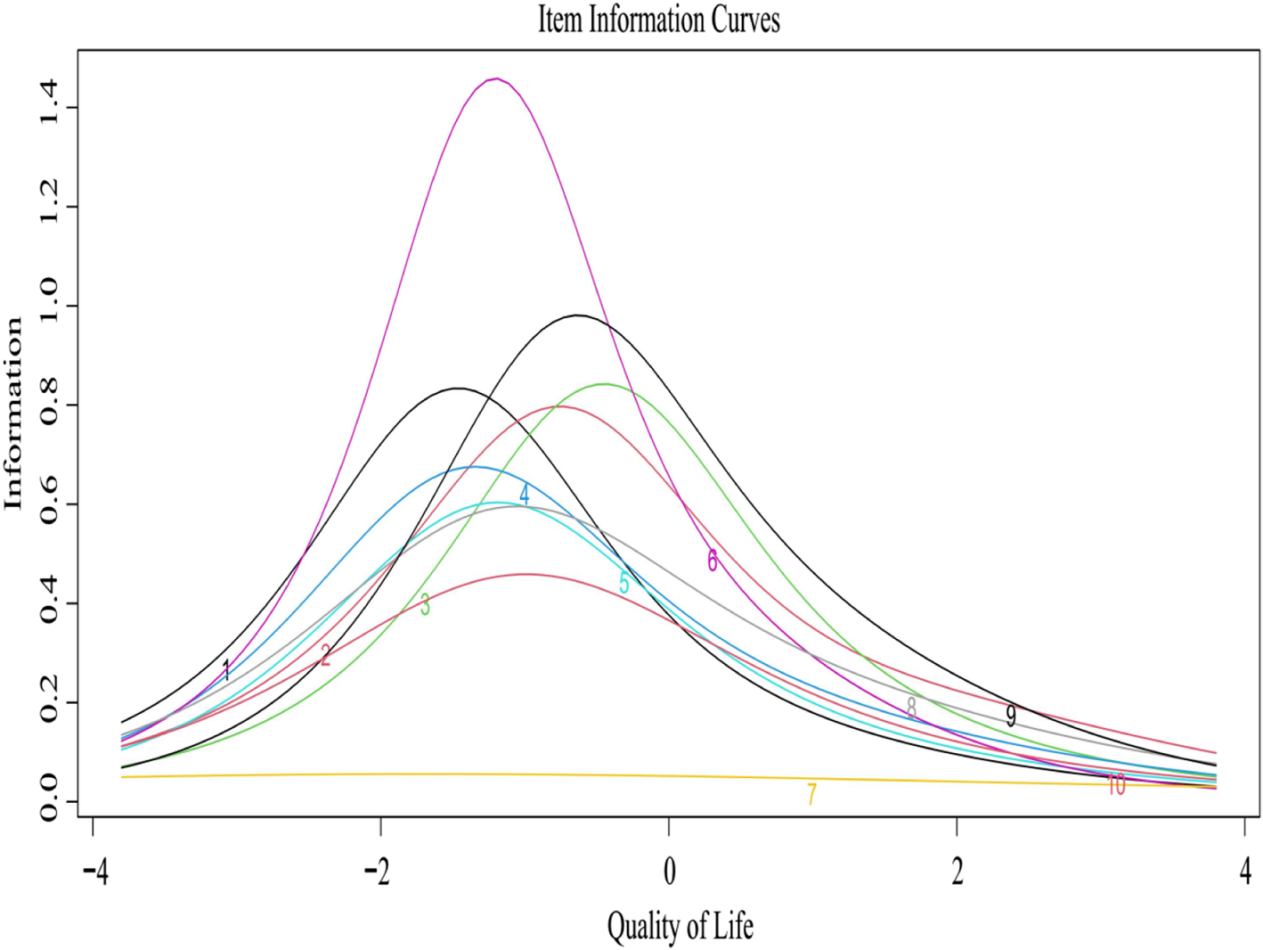
Item Information Curves for the SPD-10. Each number on the curves corresponds to the item number

Table 3 shows the selected items for each of the eight candidate short-forms, and the corresponding Cronbach alphas and concurrent validity with the SPD-10. The shortened versions with at least seven items satisfied our pre-specified criteria on reliability and concurrent validity. Figure 2 shows the individual item information curves for the SPD-10. Those for the three removed items were flat. The 7-item version, SPD-7, which included CRF1, CRF2, CRF3, CRF4, CRF6, CRF8, and CRF9, was identical to the one derived by the tandem use of GT, CTT and IRT.

**Table 3:** The selected items for short form based on OTA and their psychometric properties

Number of items	Items selected										Reliability	Concurrent validity with the SPD-10	
	CRF1	CRF2	CRF3	CRF4	CRF5	CRF6	CRF7	CRF8	CRF9	CRF10	Cronbach alpha	Correlation of the factor scores	Correlation of the total scores
3		√				√			√		0.648	0.892**	0.851**
4		√	√			√			√		0.721	0.923**	0.886**
5		√	√			√		√	√		0.733	0.952**	0.929**
6	√	√	√			√		√	√		0.758	0.962**	0.938**
7	√	√	√	√		√		√	√		0.778	0.980**	0.957**
8	√	√	√	√	√	√		√	√		0.797	0.990**	0.971**
9	√	√	√	√	√	√		√	√	√	0.808	0.999**	0.987**
10	√	√	√	√	√	√	√	√	√	√	0.795	1.000**	1.000**

**P<0.01

### Reliability of the SPD-7

Table 4 reports the reliability of the SPD-7. The Cronbach alpha of the SPD-7 was 0.78, and the alpha after deleting one item ranged from 0.74 to 0.77. The split-half reliability was 0.75. Compared with the SPD-10, the number of items in the short-form decreased by 30%, with only a 2.1% reduction in alpha and 1.4% reduction in the split-half reliability.

**Table 4:** Comparison of reliability indexes between SPD-7 and SPD-10

Scale	Number of items		Cronbach alpha		Split half reliability		Score distribution		
	Value	Variance (%)	Value	Variance (%)	Value	Variance (%)	Score	Median (*Q1, Q3*)	Mean
SPD-10	10	—	0.80	—	0.76	—	17.50 ~ 95.00	65.00 (50.00 ~80.00)	64.51
SPD-7	7	30.00	0.78	2.14	0.75	1.44	10.71 ~ 100.00	65.00 (50.89~78.57)	64.77

SPD-10:a chronic renal failure-specific module; SPD-7: a 7-item shortened form obtained. *Q1:* the item score is the 25% from small to large. *Q3:* the item score is the 75% from small to large

### Construct validity and Concurrent validity of the SPD-7

A one-factor model was fitted to the SPD-7 in a CFA. The model fit indices were TLI = 0.94 (> 0.90), CFI = 0.96 (> 0.90), and RMSEA = 0.09 (< 0.1), indicating acceptable model fit.

Moreover, the correlation of the total score of the SPD-7 with that of the full-form was *r*=0.96. The correlation of the factor scores between the full and the shortened versions was *r*=0.98. The two correlation coefficients were high [35], indicating that the SPD-7 well resembled the full-form.

### Test Information of the SPD-7

**Fig. 3:**
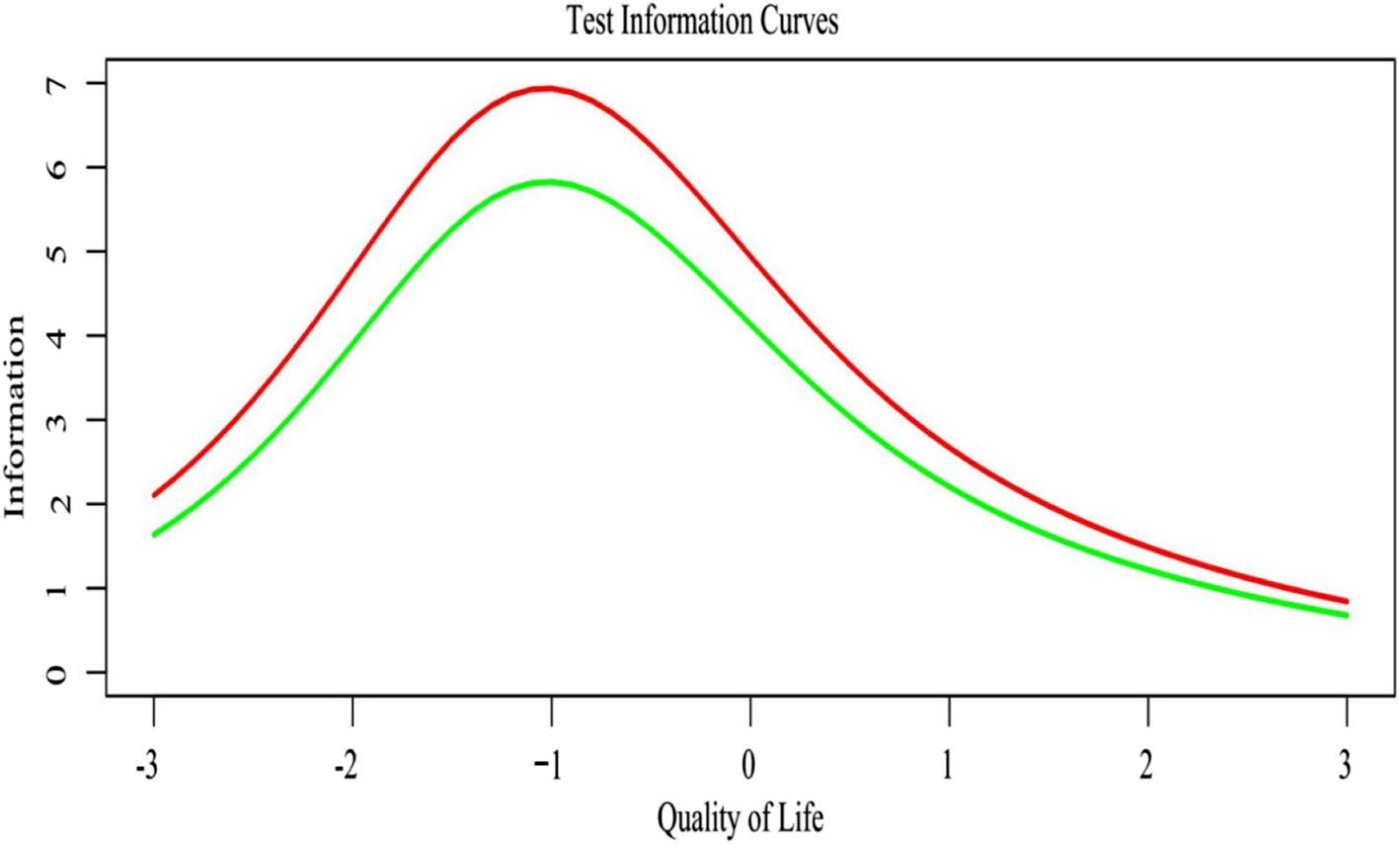
Test Information Curves for the SPD-10 (red line) and the SPD-7 (green line)

Figure 3 shows the test information over (-3, 3) of the SPD-10, and that of the SPD-7. The test information of the SPD-7 closely resembled that of the SPD-10.

## Discussions

We made the first attempt to develop a short-form of the SPD-10 for CRF patients by two approaches. The first approach utilized the GT, CTT, and IRT simultaneously. The GT analysis (D-study) showed seven items were adequate, with *G* and *ɸ* >0.7, and ${\sigma}_{\delta}^2$ and ${\sigma}_{\Delta }^2$<0.2 [10]. The availability of such thresholds enhanced the objectivity in determining the required number of items. The CTT and IRT analysis selected seven items based on those with the higher average item information, and thus contributed to a more comprehensive perspective in the scale reduction process. In the second approach, we showed how the OTA could be used to develop a short-form of the SPD-10 based on pre-specified objective criteria. Coincidentally, both the OTA and the tandem use of GT, CTT and IRT derived the same short-form, the SPD-7.

Both approaches removed items CRF7, CRF5 and CRF10. In particular, item CRF7 "Have you urinated a lot at night?" performed poorly in discrimination, validity and average information. This can be due to the fact that nocturia is one of the earliest manifestations of CRF, which most patients would ignore [36]. The other two items: CRF5 "Have you had skin itchiness?" and CRF10 "Have you had constipation?" had the lowest IIF curves, indicating the items did not add to the precision for measuring QOL [37]. Indeed, a previous study showed that most patients with CRF had pruritus [38], which is also observed in our survey. When most patients have frequent pruritus, it may not add information to distinguish CRF patients with different QOL due to pruritus. Similarly, item CRF10 on constipation also had very low information; thus, it was also not included. Thus, although the three items cover symptoms relevant to CRF patients and their removal may be seen as reducing the content validity, their high prevalence did not warrant their inclusion for discriminating the QOL of CRF patients.

Reliability analysis showed that the SPD-7’s Cronbach alpha and split-half reliability were 0.78 and 0.75, respectively, corresponding to only 2.14% and 1.44% reduction of those of the full-form. Since the commonly accepted minimum level of reliability is 0.70 [39], the SPD-7 has acceptable reliability. Moreover, the CFA confirmed the validity of the one-factor model of the SPD-7, assuring its construct validity [21]. In addition, the total and factor scores of the SPD-7 were highly associated with those of the SPD-10, implying its good concurrent validity. Furthermore, the test information curves of the SPD-7 and the SPD-10 closely resembled each other, indicating that the short-form holds similar precision to measure QOL of CRF patients at the same latent trait level [40].

Overall, when considering internal consistency, correlation of the total and factor scores, construct validity and test information, the short-form SPD-7 showed good performance without compromising the performance of the original form. Therefore, SPD-7 is a viable alternative that can improve the feasibility of assessing QOL in CRF patients.

Generally speaking, the tandem approach of using the GT, CTT and IRT selected items with better quality from different aspects, and the OTA approach selected the short-form that best resembles the full-form in selected measurement properties. Specifically, the tandem use of the GT, CTT, and IRT involved a subjective selection of items based on their IIF despite objectively determining the number of items. Thus, the approach may result in different shortened versions, when used by different researchers. On the other hand, the OTA allows objective determination of items based on pre-specified criteria. Nevertheless, both approaches arrived at the same short-form in our application and thus, the SPD-7 should be the most robustly derived.

There were several limitations worth noting. First, the sample size of this study was not very large, which may affect some results, e.g., the factor analysis and the IRT. Indeed, the IRT generally requires a large sample size for adequate stability. However, a study that investigates the effects of test length and sample size on IRT parameters found that a sample size as small as 150 could still be used with tests of 10, 20, or 30 items with enough accuracy [41]. Thus, with only 10 items in SPD-10, our sample of 164 would remain reasonable for the IRT analysis. Second, we included inpatients only, which may affect the generalizability of the results. Third, we had not evaluated the short-form in an independent sample. Future studies evaluating the SPD-7 in outpatients and inpatients through external data would be desirable.

## Conclusions

The SPD-7 can be used for assessing the impacts of clinical symptoms and side effects on QOL in CRF patients, with reliability and validity as good as its full-form. It is an efficient option, especially when used with the general module QLICD-GM for a more comprehensive QOL assessment.

## Data Availability

The datasets used and analyzed during the current study are available from the corresponding author on reasonable request.

## Abbreviations

GT: Generalizability theory
CTT: Classical test theory
IRT: Item response theory
OTA: Optimal test assembly
CRF: Chronic renal failure
QOL: Quality of life
QLICD-CRF: Quality of Life Instruments for Chronic Disease-chronic renal failure
SPD-10: a 10-item specific module for chronic renal failure
SPD-7: a 7-item short form of specific module for chronic renal failure
GPCM: Generalized partial credit model
